# Virtual Intervention to Support Self-Management of Antiretroviral Therapy Among People Living With HIV

**DOI:** 10.2196/jmir.3264

**Published:** 2015-01-06

**Authors:** José Côté, Gaston Godin, Pilar Ramirez-Garcia, Geneviève Rouleau, Anne Bourbonnais, Yann-Gaël Guéhéneuc, Cécile Tremblay, Joanne Otis

**Affiliations:** ^1^Research Center of the Centre Hospitalier de l’Université de MontréalMontreal, QCCanada; ^2^Université de MontréalFaculty of NursingMontreal, QCCanada; ^3^Research Chair in Innovative Nursing PracticesMontréal, QCCanada; ^4^Laval UniversityFaculty of NursingQuebec, QCCanada; ^5^École PolytechniqueCanada Research Chair on Software Patterns and Patterns of SoftwareMontreal, QCCanada; ^6^Immunology Laboratory at the Quebec Public Health LaboratorySainte-Anne-de-Bellevue, QCCanada; ^7^Université de MontréalFaculty of MedicineMontréal, QCCanada; ^8^Université du Québec à MontréalCanada Research Chair in Health EducationMontreal, QCCanada

**Keywords:** adherence, antiretroviral therapy, highly active, HIV-infected patients, human immunodeficiency virus, nursing, nursing informatics, Web-based intervention

## Abstract

**Background:**

Living with human immunodeficiency virus (HIV) necessitates long-term health care follow-up, particularly with respect to antiretroviral therapy (ART) management. Taking advantage of the enormous possibilities afforded by information and communication technologies (ICT), we developed a virtual nursing intervention (VIH-TAVIE) intended to empower HIV patients to manage their ART and their symptoms optimally. ICT interventions hold great promise across the entire continuum of HIV patient care but further research is needed to properly evaluate their effectiveness.

**Objective:**

The objective of the study was to compare the effectiveness of two types of follow-up—traditional and virtual—in terms of promoting ART adherence among HIV patients.

**Methods:**

A quasi-experimental study was conducted. Participants were 179 HIV patients on ART for at least 6 months, of which 99 were recruited at a site offering virtual follow-up and 80 at another site offering only traditional follow-up. The primary outcome was medication adherence and the secondary outcomes were the following cognitive and affective variables: self-efficacy, attitude toward medication intake, symptom-related discomfort, stress, and social support. These were evaluated by self-administered questionnaire at baseline (T0), and 3 (T3) and 6 months (T6) later.

**Results:**

On average, participants had been living with HIV for 14 years and had been on ART for 11 years. The groups were highly heterogeneous, differing on a number of sociodemographic dimensions: education, income, marital status, employment status, and living arrangements. Adherence at baseline was high, reaching 80% (59/74) in the traditional follow-up group and 84% (81/97) in the virtual follow-up group. A generalized estimating equations (GEE) analysis was run, controlling for sociodemographic characteristics at baseline. A time effect was detected indicating that both groups improved in adherence over time but did not differ in this regard. Improvement at 6 months was significantly greater than at 3 months in both groups. Analysis of variance revealed no significant group-by-time interaction effect on any of the secondary outcomes. A time effect was observed for the two kinds of follow-ups; both groups improved on symptom-related discomfort and social support.

**Conclusions:**

Results showed that both interventions improved adherence to ART. Thus, the two kinds of follow-up can be used to promote treatment adherence among HIV patients on ART.

## Introduction

For over a decade, HIV infection has been considered a chronic disease thanks to the advent of antiretroviral drugs [1,2]. Living with HIV necessitates long-term health care follow-up particularly with respect to management of antiretroviral therapy (ART), which today entails daily medication intake for life. The latest guidelines recommend that ART initiation be offered to all upon HIV infection diagnosis [3]. Major developments in information and communication technologies (ICT) over the years have allowed diversification of the modalities and kinds of interventions available to support ART adherence or self-management. ICT can also facilitate access to a wide variety of interventions at a favorable cost-effectiveness ratio [4].

Recent literature reviews have reported the deployment of ICT-supported interventions for HIV patients. Pellowski and Kalichman [5] reviewed 12 studies, of which nine focused on adherence to HIV treatment subsequent to interventions delivered via different technologies, including short message service (SMS)/text messaging, cell phones, and computers. The systematic review realized by Saberi and Johnson [6] turned its attention to technology-based self-care approaches likely to improve ART adherence. According to these authors, a clear pattern of results emerged in favor of individually tailored interventions that used multi-function technologies allowing for periodic communications with health care providers instead of merely relying on electronic reminder devices. Moreover, recent reviews [7,8] have suggested that ICT-assisted interventions, such as sending text messages via mobile phone, had the potential to increase treatment adherence in sub-Saharan Africa. Pellowski and Kalichman [5] suggested that technology-delivered interventions held great promise across the entire continuum of HIV patient care but that further research was needed to properly determine and assess their effectiveness.

We developed a virtual intervention called HIV Treatment, Virtual Nursing Assistance and Education or VIH-TAVIE (from its French appellation, *Virus de l’immunodéficience humaine - Traitement assistance virtuelle infirmière et enseignement*) to empower HIV patients to manage their ART and their symptoms optimally. Based on theories of behavior change [9,10], VIH-TAVIE targets the development and consolidation of skills to enhance the individual’s ability to act [11]. VIH-TAVIE consists of four interactive sessions at a computer, which are hosted by a virtual nurse who engages the user in a self-management skills-learning process [12]. Unlike conventional health-related websites that contain a library of information, VIH-TAVIE is a tailored intervention driven by a decision tree. What distinguishes this intervention from others is its mechanism of action, a tailored design that adapts content and messages to user profile and needs, the scientific rigor of its proposed content, and its flexibility of use. The intervention is the end point of a number of previous activities, which consisted of identifying predictors of treatment adherence [13], developing an intervention program aimed at promoting adherence [11], using innovative technology through the creation of a virtual intervention [12], and carrying out a preliminary validation among HIV patients [14].

As there is no cure for HIV infection, the number of chronically affected individuals is on the rise, placing growing demands on health care systems. Health resources need to be optimized in order to provide better support to service user groups living with various chronic health conditions. In this light, the aim of the present project was to compare the efficacy (value) of two kinds of health care follow-up—traditional and virtual (VIH-TAVIE)—on treatment adherence behavior among HIV patients on ART.

## Methods

### Study Design

A quasi-experimental study was conducted to evaluate the capacity of both kinds of follow-up to optimize medication adherence (primary outcome). Adherence is a behavioral indicator that can be predicted in part by cognitive and affective variables (secondary outcomes), particularly sense of self-efficacy and attitude toward drug intake [13], which in turn can be explained by perceived social support and absence of symptoms. The primary and secondary outcomes were selected on the basis of previously identified predictors of treatment adherence among HIV patients [13]. There were three measurement times: baseline (T0), 3 months (T3), and 6 months (T6) later. The study was approved by the Research Ethics Board of the Université de Montréal, the Research Centre of Centre Hospitalier de l’Université de Montréal, and the McGill University Health Centre.

### Recruitment of Participants

Participants were recruited at two university hospitals that deliver specialized care and services to HIV patients in the same metropolitan area. The two sites share the same mission of care and services, teaching, and research in the field of HIV/AIDS. The HIV patients recruited had to be at least 18 years old and on ART for at least 6 months. Pregnant women, people with uncontrolled psychiatric conditions, and active intravenous drug users were not eligible for the study. It was estimated that an initial sample size of 186 participants was needed in order to detect a difference of 20 percentage points at 80% power and an alpha level of .05 for two-tailed chi-square tests.

### Non-Random Assignment

Recruitment took place at the two university hospitals, one of which offered virtual follow-up in addition to traditional follow-up. Thus, one group was formed at each recruitment site: the traditional care group and the group with the adjunctive virtual intervention. Of the 179 participants, 99 were recruited at the site offering virtual follow-up adjunctive to traditional follow-up and 80 at the other site offering only traditional follow-up.

### Follow-Up

**Figure 1:**
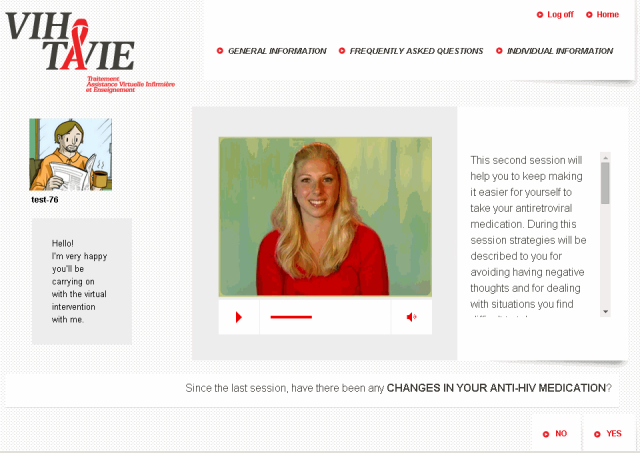
Illustration of the first page of session 2 of VIH-TAVIE.

In virtual follow-up, the four VIH-TAVIE sessions, each 20-30 minutes long, were offered over an 8-week period following the baseline measurement. Each interactive session is distinct from the other in terms of messages, strategies, skills, questions, and data entry (for example, see Figure 1).

**Figure 2:**
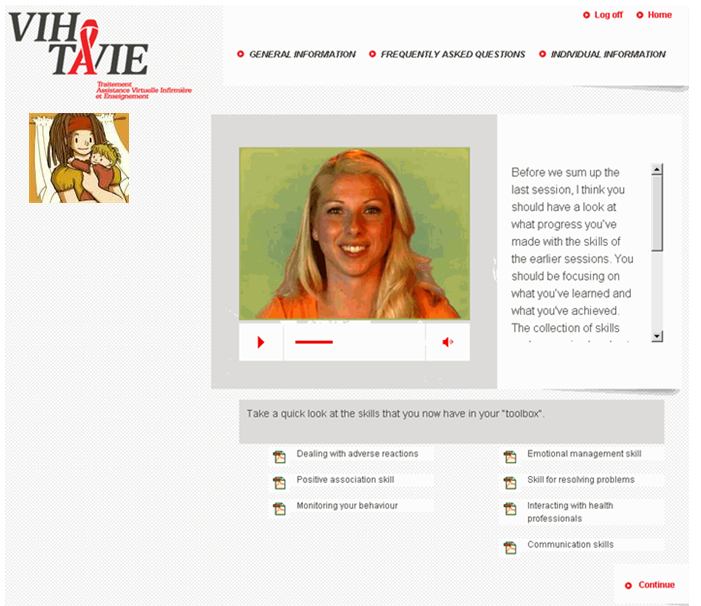
Illustration of a page of session 4 of VIH-TAVIE.

The sessions follow a predefined sequence in order to ensure a gradual conveyance of abilities. The first session focuses on developing self-assessment skills, reinforcing and developing motivational skills, such as associating a positive image with treatment, and managing undesirable and adverse effects. The second session deals with emotional management by learning to identify negative sentiments, recognizing their effects on behavior, and acquiring strategies to cope with them. It serves, also, to review the problem-solving process for dealing with situations where medication intake is awkward. The third session concerns how to establish, maintain, and strengthen social relations and interact with health professionals. In the final session, all the skills previously worked on are consolidated, as illustrated in Figure 2.

At the heart of the application is a virtual nurse who acts as coach interacting with the user asynchronously. Over the course of the sessions, approximately 140 video clips of the virtual nurse are presented, ranging in duration from 10 to 90 seconds. The virtual nurse provides feedback and positive reinforcement on the user’s personal style and methods and on skills acquired. Aside from delivering tailored teaching, the virtual nurse also refers to the experiences of other HIV patients who have been able to cope successfully with situations similar to those of the user. The originality of this Web application lies in the process of interaction with the individual, which contrasts with standard health information websites where much of the content is passively presented and interaction with the user is minimal, if not nil. Participants who benefitted from VIH-TAVIE could also consult their regular health care teams.

Traditional follow-up consisted of meeting with health care professionals over a period of 3 to 4 months. The meetings lasted 20 minutes and covered medication, symptoms, and problems encountered. Personalized health advice was given on these occasions.

### Measures

Adherence, the study’s primary outcome, was evaluated by means of a self-administered questionnaire developed and validated among HIV patients following the latest recommendations regarding the measure of therapy adherence [15]. The questionnaire comprised seven items serving to measure how often a person forgot to take their medication. It is designed to place the respondent in a context where events and situations could lead to lapses. The questionnaire’s validity was demonstrated (sensitivity: 71%; specificity: 72%; correct classification: 72%; OR 6.15) using immunologic (CD4 count) and virologic (viral load) parameters as criteria. Adherence was defined as the intake of at least 90% of prescribed tablets. Though there is no clear minimum cut-off point defining what constitutes sufficient ART adherence for optimal treatment effectiveness [16], this is generally set at >90% to >95% [17].

Self-efficacy regarding medication intake was measured with 12 items rated on a 5-point Likert scale. These items were adapted from a scale developed by Godin et al [15] and previously used on a larger sample (N=399). To adapt the instrument to the present context, that is, ART medication intake, the items were reviewed by expert consensus on the basis of the results of a focus group, a literature review, and Bandura’s theoretical model of self-efficacy. Content validation was carried out. A Cronbach alpha of .88 was obtained for this study.

Attitude toward medication intake was evaluated through six items rated on a 5-point Likert scale. These items emerged from focus groups of HIV patients on treatment. A preliminary version of the instrument was tested among 35 HIV patients. The scale was previously used on a large sample (N=399) [13], obtaining a Cronbach alpha of .83 and a test-retest reliability coefficient of .72. A Cronbach alpha of .86 was obtained for this study.

Symptom-related discomfort was measured with the Self-Completed HIV Symptom Index [18]. This 20-item instrument serves to determine whether symptoms are present on a scale of 0 to 4 (0=absence) and the degree of discomfort experienced (1, 2, 3, or 4). The instrument was validated among 188 HIV patients, demonstrating acceptable psychometric properties, including good construct validity. A Cronbach alpha of .93 was obtained for this study.

Social support was evaluated using the Medical Outcome Survey [19,20]. One dimension of social support was measured by the emotional support (8 items) subscale. Items are rated on a 5-point Likert scale. The instrument has demonstrated good content validity and appreciable internal consistency. A Cronbach alpha of .97 was obtained for this study.

Stress was measured with the stressfulness subscale (4 items) of the Stress Appraisal Measure developed by Peacock and Wong [21]. A Cronbach alpha of .90 was obtained for this study.

Immunologic and viral indicators were captured through CD4 count and viral load, respectively. This information was garnered from the participants’ medical files.

### Statistical Analyses

Descriptive statistics (frequency tables, means, and standard deviations) were summarized at each time point. Student’s *t* tests or chi-square tests were performed for each sociodemographic and baseline psychological variable to verify group equivalence. A generalized estimating equations (GEE) analysis was run to test differences in adherence over time (baseline, 3 months, and 6 months) between kinds of follow-up (traditional vs virtual: dichotomous variable). For the analysis, an intention-to-treat approach was applied. Thus, all available data for each participant were used in the analysis model. To test changes in continuous variables (attitudes, self-efficacy, symptoms, stress, and social support) over 6 months for both groups, we compared measures of change at T3 and T6 separately with two *t* tests. Then, we compared the two measures of change using a two-factor (time and group) repeated-measures analysis of variance (ANOVA). Finally, we ran the same analysis controlling for the measure at T0. In all cases, the ANOVAs were based on a generalized linear model (GLM) and no allocation technique (no imputation of missing data) was used.

## Results

### Sample Characteristics

The sample included 23 women and 153 men and had a mean age of 48 years (SD 8.4, range 23-73). On average, participants had been living with HIV for 14 years and had been on treatment for 11 years. The groups were highly heterogeneous, differing on a number of sociodemographic dimensions. For instance, compared with participants in the virtual follow-up group, those in the traditional follow-up group had more years of formal education (*P*<.001), earned more (*P*<.003), had more children (*P*<.035), and were more likely to be married (*P*<.001) and living with a spouse (*P*<.001). A high percentage of participants in each group had an undetectable viral load and maintained a CD4 level within reasonable limits. A higher proportion of participants in the virtual follow-up group reported having ceased taking anti-HIV medication in the 3 months prior to the study, compared with those in the traditional follow-up group (*P*<.030). Also, the virtual group had a lower CD4 count compared with the traditional follow-up group (*P*<.021). Table 1 presents the sociodemographic and clinical characteristics of the participants in both groups.

**Table 1:** Demographic and clinical characteristics of the participants in both groups.

Variables		Traditional follow-up (n=80)	Virtual follow-up (n=99)	*P* value
**Gender, n (%)** ^a^				.151^b^
	Female	7 (8.8)	16 (16.2)	
	Male	71 (88.8)	82 (82.8)	
Age (years), mean (SD)		49 (9.2)	47 (7.6)	.062^c^
**Ethnicity, n (%)**				<.001^b^
	Canadian	41 (51.2)	89 (89.9)	
	Other	39 (48.8)	10 (10.1)	
**Marital status, n (%)**				<.001^b^
	Single	41 (51.2)	80 (80.8)	
	Married or living as couple	31 (38.8)	7 (7.1)	
	Divorced/widowed	8 (10.0)	12 (12.1)	
**Employment status, n (%)**				<.001^b^
	Working/student	37 (46.2)	14 (14.1)	
	Insurance/retired	16 (20.0)	9 (9.1)	
	Welfare	19 (23.8)	64 (64.6)	
	Other	8 (10.0)	12 (12.1)	
**Education levels, n (%)**				.001^b^
	Primary	5 (6.2)	7 (7.1)	
	Secondary	27 (33.8)	54 (54.5)	
	College	23 (28.7)	29 (29.3)	
	University	25 (31.2)	9 (9.1)	
**Annual income, CAD, n (%)** ^a^				.003^b^
	<$14,999	33 (41.2)	67 (67.7)	
	$15,000 - $34,999	20 (25.0)	21 (21.3)	
	$35,000 - $54,999	10 (12.6)	5 (5.0)	
	>$55,000	11 (13.8)	3 (3.0)	
Years of HIV infection, mean (SD)		14.4 (7.3)	13.4 (7.7)	.365^c^
Years on antiretroviral therapy, mean (SD)		11.6 (6.6)	9.9 (6.5)	.077^c^
Treatment interruption 3 months before baseline T0, n (%)		4/79 (5.1)	15/99 (15.2)	.03^b^
Viral load less than 50 copies, n (%)		60/67 (89.6)	67/82 (81.7)	.179^b^
CD4 count (cells/μl), mean (SD)		540 (293)	441 (237)	.021^c^

^a^Subgroups do not always add up to totals owing to missing data.

^b^Chi-square test.

^c^Student’s *t*-test.

### Engagement and Participation in Virtual and Traditional Follow-Up

Of the 99 participants in the virtual follow-up group, 73 completed all four VIH-TAVIE sessions, three completed three, seven completed two, 12 completed only one, and four completed none. The computer sessions took place every 2 weeks at the hospital/clinic over a period of 6 to 8 weeks. The time required to complete session 1 varied from 20 to 30 minutes, sessions two and three took 20 minutes to complete, and session 4 required about 10 minutes. Participants in the virtual follow-up group could also consult their regular health care teams during the study’s 6-month period. Of the 80 participants in the traditional follow-up group, 60% (48/80) met with their medical teams twice and the remaining 40% (32/80) met three times.

### Effect on Adherence Behavior

Adherence at baseline was high, reaching 80% (59/74) for the traditional follow-up group and 84% (81/97) for the virtual follow-up group. Given the inter-group differences at baseline in terms of sociodemographic characteristics, a GEE analysis was run controlling for age, living alone, income, symptoms, and employment status. A time effect was observed, whereby the two groups improved on adherence over time but did not differ. Improvement at 6 months was significantly greater than at 3 months for both groups. No significant group-by-time interaction was noted. Table 2 shows the GEE results for kind of follow-up effect on medication intake adherence (≥90%).

**Table 2:** Effect of follow-up type on medication adherence (% of adherence ≥90%) using generalized estimating equations (GEE)^a^.

Type of follow-up	Baseline (T0) % adherence ≥90	3 months (T3) % adherence ≥90	6 months (T6) % adherence ≥90
Traditional follow-up (n=80)	79.7	85.7	92.7
Virtual follow-up (n=99)	83.5	90.4	89.6

^a^Group x Time interaction, *Z*=−1.36, *P*=.1743; time effect, *Z*=−1.96, *P*=.0496.

### Effect on Cognitive and Affective Variables

Overall, participants reported on symptoms in terms of quantity and perceived discomfort. They also expressed positive attitudes, a high sense of self-efficacy, a low level of stress, and a moderate level of perceived social support. Nonetheless, at baseline, the virtual follow-up group reported more symptoms, a higher level of stress, and less perceived social support, compared with the traditional follow-up group. Table 3 gives a description of the cognitive and affective variables relative to the participants in each group.

Statistical analyses revealed no significant group x time interaction on self-efficacy, attitude toward medication intake, symptom-related discomfort, stress, or social support. A time effect was observed for both kinds of follow-up on symptom-related discomfort and social support. Both groups improved over time with respect to these variables. ANOVA results regarding type of follow-up effect on cognitive and affective variables are presented in Table 4. These ANOVAs were based on a GLM with no data imputation technique used. Participants lost to attrition at T3 and T6 were considered missing at random. To verify our strategy, analyses were run to compare characteristics at T0 based on presence/absence of participants at T6. Differences emerged in terms of age, having children (yes/no), income, and ART drug intake cessation. Accordingly, there were more losses among younger participants, those with no children, those with lower income, and those who had ceased taking ART medication 3 months prior to the study. However, these differences were not of the sort to call into question the analysis strategy used. Moreover, as attrition was higher in the traditional follow-up group, use of the last observation carried forward (LOCF) allocation method would have contributed to obtain the desired results (difference of evolution between groups) by generating a larger number of stable participants in the traditional follow-up group compared with the virtual follow-up group.

**Table 3:** Cognitive and affective variables of the participants in both groups.

Cognitive and affective variables	Traditional follow-up (n=80) mean (SD)	Virtual follow-up (n=99) mean (SD)	*P* value^a^
Symptoms count^b^	9.85 (7.17)	12.57 (6.91)	.011
Symptoms bother^c^	21.16 (17.19)	29.07 (18.23)	.004
Attitude^d^	23.90 (5.32)	23.46 (4.69)	.554
Stress^e^	6.23 (3.67)	7.45 (4.04)	.037
Self-efficacy^f^	1246.25 (206.22)	1192.89 (196.89)	.079
Social support^g^	70.23 (21.61)	60.77 (20.03)	.003

^a^Student’s *t* test.

^b^Possible range 0-24.

^c^Possible range 0-96.

^d^Possible range 6-30.

^e^Possible range 4-20.

^f^Possible range 0-1400.

^g^Possible range 19-95.

**Table 4:** Effect of follow-up type on cognitive and affective variables using ANOVA.

Variables/type of follow-up		Group x time interaction *F*, *P* value	Time effect *F*, *P* value
**Symptoms count**			
	Virtual (n=67)	*F*=0.322, *P*=.572	*F*=4.166, *P*=.044
	Traditional (n=31)		
**Symptoms bother**			
	Virtual (n=67)	*F*=0.562, *P*=.455	*F*=4.127, *P*=.045
	Traditional (n=31)		
**Attitude**			
	Virtual (n=67)	*F*=3.759, *P*=.056	*F*=1.069, *P*=.304
	Traditional (n=29)		
**Stress**			
	Virtual (n=68)	*F*=0.871, *P*=.353	*F*=1.915, *P*=.170
	Traditional (n=32)		
**Self-efficacy**			
	Virtual (n=68)	*F*=0.268, *P*=.606	*F*=1.416, *P*=.237
	Traditional (n=32)		
**Social support (total score)**			
	Virtual (n=68)	*F*=0.184, *P*=.669	*F*=5.647, *P*=.019
	Traditional (n=32)		

## Discussion

### Principal Results

The objective of the study was to compare the effectiveness of two kinds of follow-up—traditional and virtual—in promoting adherence behavior among HIV patients on ART. We expected virtual follow-up to produce greater adherence on account of the use of an innovative tool, VIH-TAVIE, designed to empower HIV patients to self-manage their ART. Results showed that the two groups improved in adherence at 6 months but did not differ in this regard. Hence, neither type of follow-up proved better than the other in terms of treatment adherence.

The two groups or cohorts studied had been living with HIV for 14 years, had been under therapy for 10 years, reported many symptoms, but maintained a high adherence level that far exceeded percentage levels reported in the literature. In this regard, a recent meta-analysis of 84 studies from 20 countries [17] showed that only 62% of participants reported 90% or better ART adherence. Despite a high percentage at baseline that left little room for improvement, the two groups still managed to improve over time. The two groups also improved in terms of symptom-related discomfort and perceived social support. Sense of self-efficacy and attitude toward ART medication intake, which were high at baseline, remained unchanged.

It is worth noting that with both types of follow-up—virtual and traditional—individuals improved in adherence over the 6-month period. This is a significant clinical outcome given the consequences of non-adherence on treatment’s effectiveness to stop viral replication and the individual’s subsequent state of health. As it is documented, poor adherence remains a major risk factor for virologic failure and the development of resistance [22] and has major implications regarding future viral transmission.

Notwithstanding our results, Hersch and colleagues [23] reported that the adherence rate of participants in their Web-based adherence program did not improve but actually decreased slightly, whereas the adherence rate in the control group declined from about 85% to 66%.

Such differences in reported findings can be attributed to the difficulty of observing improvement in adherence among HIV patients. This was observed by Mathes et al [24]. In their systematic review, they pointed out that the likelihood of finding only slight differences between groups is limited by a ceiling effect (high baseline adherence) and the fact that comparison groups benefit from adherence-enhancing components in their usual follow-up. Also, the meta-analysis by de Bruin and colleagues [25] showed that standard adherence care (traditional follow-up) had a large impact on HIV treatment effectiveness and adherence. In fact, these authors found these HIV patients in the experimental and the comparison groups (standard adherence care) of ART adherence intervention trials were exposed to effective adherence care that explained up to 55% of treatment success rates.

At present, comparisons with previous studies remain difficult given the scant literature on interventions similar to VIH-TAVIE. Most of the published studies concern interventions using mobile phones [26-28] or SMS/text messaging [8,28-33]. To date, only Fisher and colleagues [34] and Hersch and colleagues [23] have evaluated Web-based HIV medication adherence interventions comparable to VIH-TAVIE. Fisher et al [34] conducted a large randomized controlled trial (RCT) to evaluate or test the efficacy of a computer-administered ART adherence support intervention. Though an intention-to-treat analysis (n=594) revealed no significant impact on medication adherence compared with usual interventions (standard care), a protocol analysis (n=328) that considered only participants who completed all planned sessions showed a significant increase in self-report adherence for the computer-based intervention group. Hersch and colleagues [23], for their part, tested a Web-based program intended to improve medication adherence among adult HIV patients. For the purpose, 168 participants were randomized into either the Web-based program or a wait-list control condition. Those who benefitted from the Web-based program had a significantly higher ART medication adherence rate than did patients in the control group over a 9-month period. As stated by the authors, the adherence rate of participants in the Web-based program did not improve, while the adherence rate of the control group declined substantially. The work conducted by León and colleagues [35] is very innovative and instructive as well. These researchers developed an Internet-based homecare model—Virtual Hospital—that covers management of chronic HIV-infected patients in full. It offers four main services: virtual consultations, telepharmacy, virtual library, and virtual community. In an RCT, 42 patients were followed through Virtual Hospital and 41 through standard care. Results showed Virtual Hospital to be a feasible and safe tool. In fact, the groups did not differ in terms of clinical parameters such as viral load, CD4 count, and treatment adherence.

Although ICT-assisted interventions have shown promise as an effective means of maintaining and improving medication adherence, more research is needed to determine their efficacy with larger trials and adequate statistical power. According to Pellowski and Kalichman [5], no definite conclusions can be drawn from existing research as only pilot studies with insufficient statistical power to detect even modest-sized effects have been carried out to date. In their systematic review of the literature, Linn et al [36] concluded that ICT-assisted interventions showed promising results as a means of enhancing patient adherence to prescribed long-term medication but since this constituted a relatively new field of endeavor, high-quality studies were required in the future to establish more robust evidence of the effectiveness of this type of intervention.

### Limitations

Our study is not without limitations. Above all, the absence of randomization and a deep selection bias led to the formation of highly heterogeneous groups in terms of sociodemographic characteristics and affective and cognitive variables. Statistical adjustments were necessary to correct for this. In addition, conservative statistical strategies were used to address the problem of attrition within the traditional follow-up group at T3 and T6. Unfortunately, randomization was not feasible in our clinical context and study setting. Given that group assignment was non-random, the risk for bias in the study was high.

### Future Research and Spin-Offs

In our study, VIH-TAVIE was evaluated in a hospital setting as an adjunct to usual care. Users did not need to own a computer in order to participate in the study as one was provided at the health care center. We thus managed to involve a wider range of participants than is normally reached by Web-based interventions. Indeed, three-quarters of our participants were in a precarious socioeconomic condition, collecting welfare benefits and living on annual income of less than CAD $15,000. We are presently conducting a large online trial to evaluate VIH-TAVIE’s capacity to optimize adherence among participants with Internet access [37]. Other applications developed on the basis of the TAVIE platform are currently under evaluation. These include TRANSPLANT-TAVIE (a virtual nurse intervention intended to support transplant recipients in managing their treatment) and TAVIE@coeur (a virtual nurse intervention intended to support persons with a heart condition in managing their treatment). SOULAGE-TAVIE has been field-tested with persons in the pre-operative phase of heart surgery to help them with pain management; results so far are very promising [38].

### Future Implications and Conclusions

We developed and tested an innovative intervention for providing follow-up care to HIV patients, particularly by helping patients manage their ART. This virtual tool is easy to implement and could constitute a complementary service in support of existing specialized services. Given the current short supply of specialized resources in health care and in the area of HIV-related services, VIH-TAVIE offers clinicians an additional instrument to support their professional practice and meet the needs of their clientele with a minimum investment in human resources. As it consists essentially of simulated interactions with a virtual nurse, all that is needed is to provide minimal remote computer support services in the event the user encounters technical problems. The computer platform affords the system administrator the flexibility to add and modify page models without always having to resort to a Web programmer. This reduces system costs and facilitates the implementation of solutions over the long term in a normal context of clinical practice.

## Abbreviations

ANOVA: analysis of variance
ART: antiretroviral therapy
GEE: generalized estimating equations
GLM: generalized linear model
HIV/AIDS: human immunodeficiency virus/acquired immune deficiency syndrome
ICT: information and communication technologies
LOCF: last observation carried forward
RCT: randomized controlled trial
SMS: short message service
VIH-TAVIE: HIV Treatment, Virtual Nursing Assistance and Education (Virus de l’immunodéficience humaine - Traitement assistance virtuelle infirmière et enseignement)
